# Safety of Transcranial Direct Current Stimulation of Frontal, Parietal, and Cerebellar Regions in Fasting Healthy Adults

**DOI:** 10.3390/bs8090081

**Published:** 2018-09-10

**Authors:** Abdullah Almousa, Reema Alajaji, Malak Alaboudi, Fahad Al-sultan, Shahid Bashir

**Affiliations:** 1 Department of Medicine, King Saud University, Riyadh, P.O. 11445, Saudi Arabia; amousaa0@gmail.com (A.A.); reema.ibrahem55@gmail.com (R.A.); malak.alaboudi@gmail.com (M.A.); Dr.fahad.2030@gmail.com (F.A.-s.); 2 Neuroscience Center, King Fahad Specialist Hospital Dammam, Dammam, P.O. 15215, Saudi Arabia

**Keywords:** non-invasive brain stimulation, safety, fasting

## Abstract

(1) Background: Transcranial direct current stimulation (tDCS) is a non-invasive brain stimulation modality that has been investigated in a large number of studies in terms of it is effects on brain function, safety of use, and future implications. The principal aim of this study was to investigate the safety of 1.5-mA tDCS of three brain areas, that is, frontal, partial, and cerebellar cortices, in fasting healthy individuals during the month of Ramadan. (2) Methods: In a single-blinded, sham-controlled study, we assessed the safety of a 20-min tDCS current (1.5 mA, 35 cm^2^) over the right frontal, parietal, and cerebellar cortex areas after 8 h of fasting in healthy right-handed adult subjects using a standard safety questionnaire. (3) Results: A total of 49 subjects completed the tDCS sessions and safety questionnaire. None of the sessions were stopped due to pain or discomfort during stimulation. Moreover, no subject experienced serious adverse events such as seizures or loss of consciousness. (4) Conclusions: There was no significant difference in the frequency or type of side effects between active and sham stimulation sessions. The tDCS protocol applied in this study was found to be safe in fasting healthy adults.

## 1. Introduction

Transcranial direct current stimulation (tDCS) is a non-invasive brain stimulation method that delivers a constant and direct low-amplitude electric current via electrodes placed on the scalp [1,2,3,4]. tDCS has been increasingly used in neuroscience research by a number of different investigations, either to locate specific tasks in specific brain regions [4,5,6,7,8,9], or to evaluate possible therapeutic effects in some neurological and psychiatric disorders [8,10,11,12,13]. tDCS typically employs electric currents with amplitudes ranging from 0 to 2 mA, as safety protocols generally restrict the maximum acceptable amplitude for use in human participants to 2 mA [2,3,4,11,14,15,16]. tDCS either enhances or depresses cell membrane excitability by using a positive or negative current from an anode or cathode, respectively [3,17]. Despite rapid advancements in diverse applications of tDCS in basic and clinical neuroscience in the last decade, there is an increasing need to evaluate its safety in local populations and provide feedback to regulate its use by practitioners, patients, and regulatory agencies [2,3,4,11,15,16,17,18].

A recent review on tDCS safety reported no evidence of serious adverse effects or irreversible injury produced by conventional tDCS protocols within a wide range of stimulation parameters from over 33,000 tDCS sessions and more than 1000 subjects receiving repeated sessions [18,19]. Few minor side effects are associated with tDCS, which include skin irritation, phosphenes at the start of stimulation, nausea, headaches, dizziness, and itching under the electrode. Additionally, experimental tDCS is not advised for use in individuals suffering from seizures or migraines [11,16,18]. Moreover, the safety of tDCS was proven in controlled human trials, and the mild skin erythema that occurs during the procedure is not inherently hazardous and resolves after the stimulation [19].

Three major areas of the brain (i.e., frontal, parietal, and cerebellar) have been commonly targeted for tDCS stimulation in the literature [2,4,6,19]. However, no study has examined the safety of tDCS in fasting individuals.

Owing to the extensive use of tDCS as a clinical treatment aid, it is imperative to investigate and publish additional reports on its safety and tolerability during fasting, which could aid in future studies and therapeutic uses conducted under this condition. We aimed to assess the safety and tolerability of anodal/sham tDCS in healthy adults fasting for 8 h or longer.

## 2. Materials and Methods

### 2.1. Participants

We selected 49 participants (aged 18 to 60 years) via simple random sampling from the local community during the month of Ramadan. The minimum fasting time to participate in the experiment was 8 h. All participants had normal or corrected-to-normal visual acuity, and were naive to brain stimulation. Written informed consent was obtained from each subject before the study. The study was approved by the Institutional Review Board (IRB) in King Khalid University Hospital (KKUH). Only subjects with no history of neurological or psychological disorders, epilepsy or family history of epilepsy, metal instrument inserted in the head, migraine, stroke, or any previous head surgery were included. Participants were excluded if they presented with any skin disorder at or near stimulation locations (where electrodes are placed), such as eczema, rash, or other skin conditions.

### 2.2. Procedure

Before committing to the experiment, all participants were screened to ensure that they met all of the participation criteria. Each participant underwent three tDCS sessions separated by a 1-week washout interval. Each participant was randomly assigned to the placement of two electrodes on one of the three brain areas for anodal stimulation (i.e., right dorsolateral prefrontal cortex (DLPFC), right posterior parietal cortex, and right cerebellum). For the right DLPFC, the anode was placed over scalp location F4 and the cathode over Fp1 [20]. For the right cerebellum, the overlying scalp area was localized using the international electroencephalographic 10–20 system. For cerebellar cortex stimulation, the anodal electrode was placed over the scalp area estimated to overlie the right cerebellar cortex (3 cm lateral to the inion), and the reference electrode was positioned on the skin area overlying the right buccinator muscle [21]. This method of localizing the right cerebellum has been found to be appropriate for tDCS of this area. In order to activate the right posterior parietal cortex (PPC), the anodal electrode was placed over P4 and the cathodal electrode was attached to the contralateral supraorbital area [22]. 

The current (1.5 mA) was administered for 20 min via two saline-soaked, 35-cm^2^ (Soterix Medical Inc. EasyPAD) sponge electrodes (current density of approximately 0.08 mA/cm^2^) and secured using Velcro straps, and stimulation was generated with a Soterix (Soterix Medical Inc., New York, NY, USA). A sham (control) group was also established for each stimulation area in which the electrodes were set up using either the right DLPFC, cerebellum, or PPC montage. For this group, the current was ramped up to 1.5 mA over 30 s and then ramped back down both at the beginning and end of the 20-min period.

After the 20-min session, the electrodes were removed and a physical assessment of the skin and scalp was conducted. A side effect questionnaire using a Likert scale (0–5) was administered before and after each session. Each participant was asked to respond to questions about any prevalent side effects and rate them based on severity (Tingling, Itching, Burning, Headache, Fatigue, Difficulty in concentration, Sudden mood change, Change in visual perception, Unpleasant sensation, Unpleasant sensation in vision, Nausea, Drowsiness , Feeling stimulation Right, Feeling stimulation left or burning under the electrodes). Each potential adverse event was rated from 1–5 (1 = very mild**/**5 = very severe) [23]. All the questions were translated into Arabic.

### 2.3. Data Analysis

The collected data were qualitative and the assumption of expected frequency being <20% was not violated for tingling, itching, burning, headache, or feeling the stimulation on the right side after taking off the electrodes. We used Pearson’s chi-square test for comparing the presence of these side effects before and after stimulation. As the expected frequency assumption was violated for fatigue, difficulty in concentration, acute mode change, change in visual perception, unpleasant sensation, unpleasant sensation in vision, nausea, drowsiness, and feeling the stimulation on the right side after taking off the electrodes, we used Fisher’s exact test for these side effects. Data were analyzed using SPSS (IBM SPSS Statistics for Windows, Version 21.0. IBM Corp.: Armonk, NY, USA).

## 3. Results

The current study enrolled a total of 49 participants (males: 32, 65.3%; females: 17, 34.7%); the mean age for males and females was 23.3 and 26.4 years, respectively.

No session was interrupted or stopped due to pain or discomfort during stimulation. No serious adverse events such as seizure or loss of consciousness were observed.

### 3.1. Cerebellum

The most commonly reported side effects in the active group were burning (*p* = 0.063), difficulty in concentration (*p* = 0.241), sudden mood change (*p* = 0.082), and unpleasant sensation in vision (*p* = 0.224) after anodal cerebellum stimulation. On the other hand, the following adverse effects were reported most frequently in the sham group: fatigue (*p* = 0.695), difficulty in concentration (*p* = 0.241), unpleasant sensation in vision (*p* = 0.224), and drowsiness (*p* = 1.00) (Table 1).

The most severe symptoms reported were fatigue in the active group (mean = 1.65, *p* = 0.121; Table 2) and sudden mood change in the sham group (mean = 1.11 *p* = 0.854). However, tingling and itching were significantly higher in the active group (*p* = 0.023 and *p* = 0.049, respectively; Table 2).

### 3.2. Dorsolateral Prefrontal Cortex (DLPFC)

The highest frequencies of side effects were reported in the active group but the difference with the sham group did not reach significance (headache *p* = 0.144, unpleasant sensation *p* = 0.271). However, less adverse effects were reported in the sham group (itching *n* = 2, *p* = 1.00; burning *n* = 2, *p* = 1.00; headache *n* = 2, *p* = 0.144; unpleasant sensation *n* = 2, *p* = 0.271; Table 3).

The severity of each adverse effect compared between the active and sham groups after the tDCS of the DLPC is shown in Table 4. The most commonly reported symptom in the active group was itching (mean = 1.13, *p* = 0.088), and change in visual perception in the sham group (mean = 1.60, *p* = 0.094; Table 4).

### 3.3. Posterior Parietal Cortex (PPC)

Table 5 shows that the most frequent side effects were reported in the active group but the difference with the sham group did not reach significance (tingling *n* = 3, *p* = 0.072; sudden mood change *n* = 5 , *p* = 0.092), and the participants in the sham group reported more side effects compared to the active group (tingling *n* = 4, *p* = 0.072; headache *n* = 4, *p* = 0.144). Table 6 shows the severity of each side effect compared between active and sham group after the tDCS session of the PPC. We found that itching was the most severe symptom in the active group (mean = 1.6, *p* = 0.77), and difficulty in concentration was the most severe in the sham group (mean = 1.7, *p* = 0.426).

## 4. Discussion

The current study was conducted to evaluate the safety of a tDCS protocol on the frontal, parietal, and cerebral regions in fasting healthy adults.

The adverse effects most commonly reported are mild headache, tingling, itching, burning sensation, and skin redness under the area of electrodes [2,3,11,12,19,24]. Our results confirm these findings; however, we also found a low frequency of these adverse effects. In our study, we did not find a significant difference in the amount of side effects reported between the active and sham stimulation groups for any of the interventions.

Our study showed that non-invasive brain stimulation with tDCS using a constant current of 1.5 mA applied over the right DLPFC, PPC, and cerebellum was not associated with safety issues after stimulation. This is in agreement with previous work [1,2,3,5,7,10,11,12,13,16]. In addition, there were no serious adverse effects such as seizures or serious headaches during or after the stimulation. None of the subjects requested to interrupt the stimulation or needed any medical intervention during or after the end of stimulation.

The safety of the application of tDCS in humans has been addressed and tested in multiple studies [1,2,6,7,8,11,12,14,15,16,24] using different safety parameters such as use of water-soaked sponge electrodes, size of electrodes, and intensity and duration of stimulation. These studies concluded that the application a direct current (DC) over the scalp does not induce negative effects.

This study demonstrated that the tDCS protocol was safe to apply in fasting healthy adults. Other studies that investigated the safety of tDCS have also demonstrated its safety in healthy adults [12,25]. The latest review of evidence on the safety of tDCS has shown no reports of serious injuries or adverse effect in humans [19]. Moreover, tDCS appears to be safe to use in healthy adults and children, obese adults, stroke patients, and individuals with mood disorders [19].

Our study confirms that tDCS is safe and can be used in fasting healthy adults as well. The common adverse effects were mild skin irritation and redness [26]. Adverse events such as tingling, itching, and burning sensation on the stimulation site occurred in the sham group at a lesser rate. This could be due to the ten seconds stimulation (ramp-up) time in the sham group. Fatigue was also reported during these sessions, which could be due to the prolonged duration of the stimulation and is thus not a direct effect of the electric stimulation.

The current study has limited generalizability due to the relatively small sample size, which may be inadequate to detect the statistical significance of some effects. Further studies with a larger sample size including other comorbities are necessary to confirm the efficacy and safety of tDCS of the frontal, parietal, and cerebellar regions of the brain. Since Ramadan fasting involves total abstinence from not only food but also fluids, the serum osmolarity and intracellular water content, and eventually the quantitative values of brain structures may have been altered. However, in the current study, we did not examine the effect of dehydration using a direct measure of body water content such as serum osmolality.

## 5. Conclusions

In conclusion, tDCS was found to be safe for use in fasting healthy adults on the frontal, parietal, and cerebellar regions of the brain. No significant difference was found in the frequency of side effects between the active and sham stimulation sessions for any of the interventions.

## Figures and Tables

**Table 1:** Number of occurrences of side effects between active and sham groups for the cerebellum.

	Total (*n* = 17)	Condition		*p*-Value
		Active (*n* = 8)	Sham (*n* = 9)	
Tingling *	4	3	1	0.077
Itching *	5	4	1	0.312
Burning *	7	5	2	0.063
Headache *	3	2	1	0.224
Fatigue **	7	4	3	0.695
Difficulty in concentration **	8	5	3	0.241
Sudden mood change **	7	5	2	0.082
Change in visual perception **	0	0	0	1.000
Unpleasant sensation **	5	4	1	0.372
Unpleasant sensation in vision **	8	5	3	0.224
Nausea **	4	2	2	1.000
Drowsiness **	6	3	3	1.000
Feeling stimulation R **	4	2	2	1.000
Feeling stimulation L *	3	2	1	0.164

* Pearson Chi-Square Test; ** Fisher’s Exact Test; R: Right; L: Left.

**Table 2:** Comparing the severity (out of 5) of side effects between active and sham groups for the cerebellum.

	Total Mean	Condition		*p*-Value
		Active (Mean)	Sham (Mean)	
Tingling	0.54	0.75	0.33	0.023 *
Itching	0.72	1.12	0.33	0.049 *
Burning	0.88	1.00	0.77	0.125
Headache	0.83	1.00	0.66	0.312
Fatigue	1.21	1.65	0.77	0.121
Difficulty in concentration	0.8	0.5	0.33	0.439
Sudden mood change	1.16	1.12	1.11	0.854
Change in visual perception	1.13	1.5	0.77	0.221
Unpleasant sensation	0.86	1.62	0.11	0.082
Unpleasant sensation in vision	0.72	1.12	0.33	0.324
Nausea	0.12	0.25	0	0.114
Drowsiness	0.36	0.62	0.11	0.087

Values are out of a total of 5; * *p*-value < 0.05.

**Table 3:** Number of occurrences of side effects between active and sham groups for the DLPFC (dorsolateral prefrontal cortex).

	Total (*n* = 18)	Condition		*p*-Value
		Active (*n* = 8)	Sham (*n* = 10)	
Tingling *	1	1	0	0.742
Itching *	4	2	2	1.000
Burning *	4	2	2	1.000
Headache *	5	3	2	0.144
Fatigue **	2	1	1	1.000
Difficulty in concentration **	2	1	1	1.000
Sudden mood change **	3	2	1	0.092
Change in visual perception **	0	0	0	1.000
Unpleasant sensation **	5	3	2	0.271
Unpleasant sensation in vision **	2	1	1	0.824
Nausea **	1	1	1	1.000
Drowsiness **	2	1	1	1.000
Feeling stimulation R **	0	0	0	1.000
Feeling stimulation L *	3	2	1	0.211

* Pearson Chi-Square Test; ** Fisher’s Exact Test; R: Right; L: Left.

**Table 4:** Comparing the severity (out of 5) of side effects between active and sham groups for the DLPFC (dorsolateral prefrontal cortex).

	Total Mean	Condition		*p*-Value
		Active (Mean)	Sham (Mean)	
Tingling	0.30	0.50	0.10	0.623
Itching	0.94	1.13	0.75	0.088
Burning	0.18	0.37	0.00	0.142
Headache	0.16	0.12	0.2	0.238
Fatigue	1.00	1	1	0.121
Difficulty in concentration	0.48	0.37	0.60	0.122
Sudden mood change	0.57	0.75	0.40	0.142
Change in visual perception	1.40	1.2	1.6	0.094
Unpleasant sensation	0.86	0.75	0.60	0.216
Unpleasant sensation in vision	0.67	1.12	0.33	0.228
Nausea	0.21	0.12	0.30	0.414
Drowsiness	0.75	0.60	0.90	0.156

Values are out of a total of 5.

**Table 5:** Number of occurrences of side effects between active and sham groups for the PPC (posterior parietal cortex).

	Total (*n* = 14)	Condition		*p*-Value
		Active (*n* = 5)	Sham (*n* = 9)	
Tingling *	7	3	4	0.072
Itching *	3	2	1	1.000
Burning *	5	3	2	1.000
Headache *	6	2	4	0.144
Fatigue **	2	1	1	1.000
Difficulty in concentration **	2	1	1	1.000
Sudden mood change **	5	2	3	0.092
Change in visual perception **	4	2	2	1.000
Unpleasant sensation **	5	2	3	0.271
Unpleasant sensation in vision **	2	1	1	1.000
Nausea **	2	1	1	1.000
Drowsiness **	3	1	2	0.842
Feeling stimulation R **	3	1	2	0.842
Feeling stimulation L *	4	2	2	1.000

* Pearson Chi-Square Test; ** Fisher’s Exact Test.

**Table 6:** Comparing the severity (out of 5) of side effects between active and sham groups for the PPC (posterior parietal cortex).

	Total Mean	Condition		*p*-Value
		Active (Mean)	Sham (Mean)	
Tingling	0.60	0.8	0.4	0.062
Itching	1.18	1.6	0.77	0.076
Burning	0.30	0.4	0.2	0.162
Headache	0.72	1.00	0.44	0.122
Fatigue	0.66	0.88	0.44	0.106
Difficulty in concentration	1.55	1.4	1.7	0.426
Sudden mood change	1.14	1.4	0.88	0.0822
Change in visual perception	0.41	0.60	0.222	0.144
Unpleasant sensation	0.36	0.40	0.33	0.622
Unpleasant sensation in vision	1.33	1.33	1.33	1.000
Nausea	0.31	0.22	0.40	0.226
Drowsiness	1.15	1.2	1.11	0.622

Values are out of a total of 5.
